# HIV point of care diagnosis: preventing misdiagnosis experience from a pilot of rapid test algorithm implementation in selected communes in Vietnam

**DOI:** 10.7448/IAS.20.7.21752

**Published:** 2017-08-29

**Authors:** Van Thi Thuy Nguyen, Susan Best, Hong Thang Pham, Thi Xuan Lien Troung, Thi Thanh Ha Hoang, Kim Wilson, Thi Hong Hanh Ngo, Xuan Chien, Kim Anh Lai, Duc Duong Bui, Masaya Kato

**Affiliations:** ^a^ World Health Organization, Vietnam Country Office, Hanoi, Vietnam; ^b^ Australian National Serology Reference Laboratory, Melbourne, Australia; ^c^ HIV Department, National Institute for Hygiene and Epidemiology, Hanoi, Vietnam; ^d^ Pasteur Institute, Ho chi Minh City, Vietnam; ^e^ HIV Laboratory, Dien Bien Provincial AIDS Centre, Vietnam; ^f^ Can Tho Preventive Medicine Centre, Can Tho City, Vietnam; ^g^ Viet Nam Authority for HIV/AIDS Control, Ministry of Health, Hanoi, Vietnam

**Keywords:** HIV testing, rapid test, algorithm, decentralization, misdiagnosis, quality assurance, Vietnam

## Abstract

**Introduction**: In Vietnam, HIV testing services had been available only at provincial and district health facilities, but not at the primary health facilities. Consequently, access to HIV testing services had been limited especially in rural areas. In 2012, Vietnam piloted decentralization and integration of HIV services at commune health stations (CHSs). As a part of this pilot, a three-rapid test algorithm was introduced at CHSs. The objective of this study was to assess the performance of a three-rapid test algorithm and the implementation of quality assurance measures to prevent misdiagnosis, at primary health facilities.

**Methods**: The three-rapid test algorithm (Determine HIV-1/2, followed by ACON HIV 1/2 and DoubleCheckGold HIV 1&2 in parallel) was piloted at CHSs from August 2012 to December 2013. Commune health staff were trained to perform HIV testing. Specimens from CHSs were sent to the provincial confirmatory laboratory (PCL) for confirmatory and validation testing. Quality assurance measures were undertaken including training, competency assessment, field technical assistance, supervision and monitoring and external quality assessment (EQA). Data on HIV testing were collected from the testing logbooks at commune and provincial facilities. Descriptive analysis was conducted. Sensitivity and specificity of the rapid testing algorithm were calculated.

**Results**: A total of 1,373 people received HIV testing and counselling (HTC) at CHSs. Eighty people were diagnosed with HIV infection (5.8%). The 755/1244 specimens reported as HIV negative at the CHS were sent to PCL and confirmed as negative, and all 80 specimens reported as HIV positive at CHS were confirmed as positive at the PCL. Forty-nine specimens that were reactive with Determine but negative with ACON and DoubleCheckGold at the CHSs were confirmed negative at the PCL. The results show this rapid test algorithm to be 100% sensitive and 100% specific. Of 21 CHSs that received two rounds of EQA panels, 20 CHSs submitted accurate results.

**Conclusions**: Decentralization of HIV confirmatory testing to CHS is feasible in Vietnam. The results obtained from this pilot provided strong evidence of the feasibility of HIV testing at primary health facilities. Quality assurance measures including training, competency assessment, regular monitoring and supervision and an EQA scheme are essential for prevention of misdiagnosis.

## Introduction

There were an estimated 260,000 people living with HIV (PLHIV) in Vietnam at the end of 2015, and the HIV epidemic is concentrated in key populations – people who inject drugs (PWID), female sex workers (FSW) and men who have sex with men (MSM) [1]. Among them, 106,373 received antiretroviral therapy (ART) accounting for 42% of PLHIV [1]. More than 50% of PLHIV have not accessed ART or have not been diagnosed. Although, HIV testing and counselling (HTC) has been expanded, especially in high and medium burden provinces, the access by key populations to this service is still limited. In 2015, the percentage of PWID, MSM and FSW who received an HIV test in the preceding 12 months was 30%, 32% and 41%, respectively [1].

Several reasons may contribute to low uptake of HTC among key populations and pregnant women including stigma, discrimination, and inconvenience or difficulties in reaching HTC facilities. In Vietnam, HTC had been mainly available at the district level facilities via client-initiated (e.g. voluntary HTC services) and provider-initiated approaches (e.g. antennal care services). To confirm a blood specimen as HIV positive, one showing reactivity in the screening test needs to be sent to a provincial-level laboratory that is authorized by the Ministry of Health (MoH) as a confirmatory testing laboratory. As a consequence, turnaround time for the results may take from one week to four weeks especially in remote and mountainous provinces. Due to limited services and long turnaround time for results and other reasons such as poor linkages between HTC and ART services sites and lack of effective monitoring of referral services, loss to follow-up between diagnosis and enrolment in care has been a significant programmatic issue in HIV cascades in Vietnam.

In the past years, HIV diagnostic rapid tests have become widely available and have good performance compared with EIA [2,3]. This allows decentralization of HIV testing from provincial and district laboratories to primary healthcare facilities. Decentralization and integration of HTC to lower-level healthcare facilities could facilitate access especially for key populations in remote and mountainous provinces. To improve access to HTC, HIV services, including same-day test results, need to be more accessible to key populations.

Vietnam is one of the few countries in the world piloting the Treatment 2.0 initiative launched by WHO and UNAIDS in 2011 [4]. One of the five pillars of this initiative is using point of care (POC) diagnosis. The pilot study was implemented in two provinces, one in the urban area of the south (Can Tho city) and one in the mountainous area of the north (Dien Bien province). Within the scope of the pilot, POC HIV testing was introduced and promoted through decentralizing and integrating HIV testing into commune health stations (CHSs). This innovative model is expected to facilitate early diagnosis and early access to ART for key populations. This pilot was designed to demonstrate and assess the performance of decentralized HIV screening and confirmatory testing, and validation in the field, of a rapid testing algorithm. This paper is based on the data reported during the Treatment 2.0 pilot.

## Methods

### Description of the pilot

#### Pilot sites

The pilot was carried out in 21 communes in seven districts: four districts in Dien Bien province (Dien Bien city, Dien Bien district, Muong Ang and Tuan Giao) and three districts in Can Tho city (Ninh Kieu, O Mon and Vinh Thanh) in August 2012. This study was part of the pilot implementation which was evaluated from August 2012 to December 2013.

#### Selection of rapid test algorithm

Based on the results of the evaluation of HIV test kits conducted by the National Institute of Hygiene and Epidemiology (NIHE) in 2011 [5], with technical assistance from the National Serology Reference Laboratory, Australia, the national technical working group selected three rapid tests to combine into an algorithm. The rapid tests included Determine HIV-1/2 (Alere, Japan) as the screening test and the ACON HIV 1/2 (ACON Laboratories, Sadiego, United States) and DoubleCheckGold HIV 1&2 (Orgenics Ltd.,Yavne, Israel) as the second and the third assays (sensitivity and specificity of these three rapid tests are presented in Table 1). The algorithm was chosen according to WHO recommendations for developing an HIV testing algorithm for diagnosis and in line with recommended sensitivities and specificities for screening and confirmatory tests [6,7].

**Table 1.** Sensitivity and specificity of the three rapid tests based on results of the national evaluation of HIV test kits

Test kits	Sensitivity (95% CI)	Specificity (95% CI)
Determine HIV-1/2	99.50 (98.94–100.0)	95.74 (94.12–97.36)
ACON HIV 1/2	99.50 (98.94–100.0)	100.0 (100.0–100.0)
DoubleCheckGold HIV 1&2	99.00 (98.20–99.80)	99.75 (99.35–100.0)

#### Determination of serostatus at commune health station

At commune health stations, trained staff provided pre-test counselling to clients and verbal consent was obtained. Between August 2012 and July 2013, venous blood was taken and plasma was collected for HIV testing. However, from August 2013 commune health staff were trained on performing finger prick blood collection and henceforth, capillary whole blood was used for the screening test. Clients whose specimens were reactive on the Determine had venous blood collected from which the plasma was extracted and sent to the Provincial Confirmatory Laboratory (PCL) for confirmation of the HIV status of such specimens, in line with the Vietnam MoH regulation on HIV testing.

**Figure 1.**
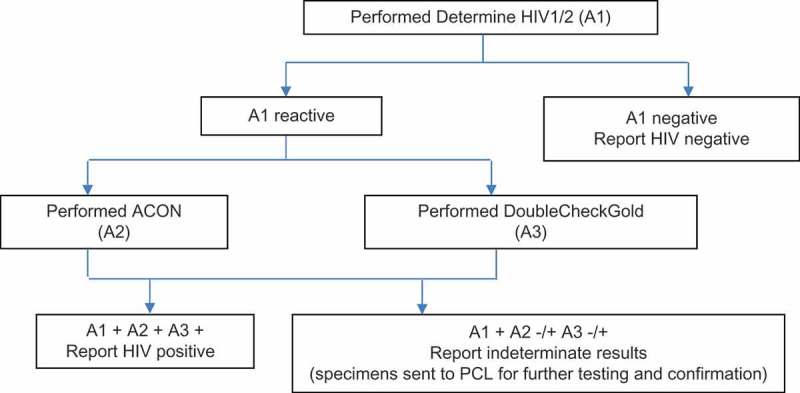
HIV testing algorithm performed at commune health stations.

For the purpose of validating the algorithm, plasma was also used for further testing with the ACON and DoubleCheckGold in parallel at the CHSs. Results of the tests were recorded in a logbook along with an overall interpretation based on the results of all tests performed. Specimens that gave non-reactive results on Determine HIV1/2 were recorded as negative; specimens that were reactive with all three tests were recorded as HIV positive; specimens that were reactive with one or two tests were recorded as indeterminate at the CHSs (Figure 1). Results for specimens showing reactivity on Determine HIV1/2 were not returned to the clients until the confirmatory test result was confirmed by the PCL. Worksheets were used to record results when performing the tests and the results of tests were then recorded in a logbook and the worksheets were sent to the reference laboratories along with the sample.

#### Validation and confirmation of HIV testing at provincial confirmatory laboratories

**Figure 2.**
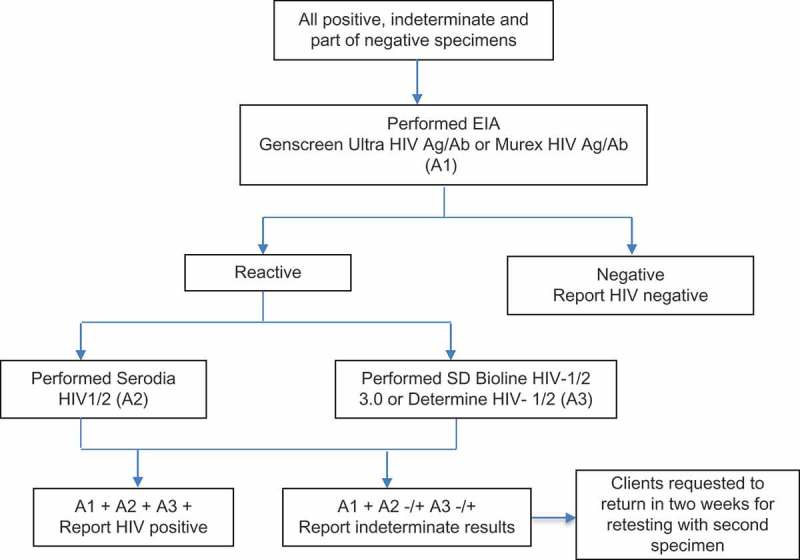
HIV testing algorithm performed at provincial confirmatory laboratory.

All specimens that were positive or indeterminate were sent to the supervising PCL for confirmation. In addition, specimens that were negative in the first three months of the pilot implementation were sent to the supervising PCL for validation. Algorithms used at both PCLs included 4th generation enzyme immunoassays (EIA), either Murex HIV Ag/Ab Combination (DiaSorin, Italy) [Murex]) or Genscreen Ultra HIV Ag/Ab EIA (Bio-Rad, France) [Genscreen] as assay one (A1), a particle agglutination assay Serodia HIV1/2 (Fujirebio, Japan) as assay two (A2), and a rapid test either SD Bioline HIV-1/2 3.0 (Alere, Korea) or Determine HIV-1/2 as assays three (A3) . For the purposes of the validation, specimens negative on Determine HIV1/2 at the CHSs were retested by Murex or Genscreen EIA. Specimens that gave negative results on EIA were confirmed as negative. Specimens that were reactive in all three tests of the respective PCL algorithm were confirmed as positive. If specimens reactive in one or two tests of the respective PCL algorithm they were given a status of “indeterminate” and the clients were requested to return into two weeks to retest (Figure 2).

Sensitivity and specificity of three rapid test algorithm was calculated based on the following formula:

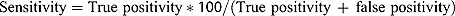

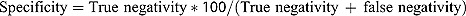

## Result communication

Clients were asked to wait for 30 min to receive results or return any time within that day according to the client’s preference. If the screening test was negative, commune health staff provided the test result and post-test counselling. In the case of reactive results, clients were counselled on the need for confirmatory testing. An appointment was made to come back to receive the confirmatory test result and referral to care if necessary. During the validation process, effort was made to expedite the confirmatory testing to ensure the clients receive the confirmatory results within 3–5 days. Prevention interventions were also encouraged during pre-and post-test counselling of the clients.

### Quality assurance

To ensure the quality of the HTC, a range of activities were undertaken including training, competency assessment, field technical assistance, supervision and monitoring and external quality assessment (EQA). Prior to the implementation of the pilot study, staff from the 21 CHS undertook a 5-day training course on HTC, delivered by trainers from NIHE and the Pasteur Institute (PI) in Ho Chi Minh City and provincial AIDS centres (PACs). In addition, a coded set of specimens was sent to each CHS for final assessment of staff competence. Only commune health staff who received certificates from training and were assessed as competent were assigned to perform HIV testing. Regular supervision and monitoring at CHS were conducted by staff from PACs and district health centres with technical assistance from NIHE, PI Ho Chi Minh City and WHO. In addition, these communes also participated in an EQA scheme. EQA panels which included 10 coded specimens were sent to these communes by NIHE twice a year to monitor the quality of HIV testing.

### Data collection and data analysis

Data on HIV testing were collected using a customized form including patient code, address, sex, year of birth and results of HIV testing at the commune and provincial levels. In addition, information on a client’s self-reported HIV risk was recorded, as PWID, MSM, sex workers, sexual partners of PWID or PLHIV and pregnant women in HTC logbooks at the CHSs and confirmatory laboratories. Descriptive analysis was conducted. Sensitivity and specificity of the rapid testing algorithm were also calculated.

### Ethical approval

The pilot was implemented following the Decision of Viet Nam Ministry of Health (Decision 1039, dated April 3 2012).

## Results

### Characteristics of clients who received HTC at CHS

Between August 2012 and December 2013, a total of 1,373 people received HTC at CHS including 938 pregnant women (68.3%), 137 PWID (10.0%), 12 FSW (0.9%), 170 partners of PWID or partners of PLHIV (12.4%) and 116 others (8.4%). Female clients accounted for 84% and most of them were pregnant women (82%) (Table 2). Eighty people were diagnosed with HIV infection (5.8%) including 6 pregnant women. All of these clients were followed and 65 were enrolled in care (81.2%), 5 died, 3 did not comeback for results, 4 moved out of province for work, 1 was sent to prison and 2 were lost-to-follow-up.

**Table 2.** Characteristics of clients who received HTC at 7 districts between August 2012 and December 2013

Characteristics	Frequency (N = 1373)	Percentage
**Age (mean±SD 28.6 ± 7.7; range 5 – 66)**		
<15	5	0.36
15–49	1336	97.3
>49	32	2.3
**Sex^a^**		
Female	1149	83.7
Male	223	16.3
**Population groups**		
Pregnant women	938	68.3
People who inject drugs (PWID)	137	10.0
Female sex workers	12	0.9
Partners of PLHIV or PWID	170	12.4
Other	116	8.4
**Residency**		
*Dien Bien province*		
Dien Bien district	386	28.1
Dien Bien city	162	11.8
Muong Ang district	214	15.6
Tuan giao district	108	7.9
*Can Tho province*		
Ninh Kieu district	230	16.8
O mon	168	12.2
Vinh Thanh district	105	7.6

^a^One missing value

### Testing algorithm validation

**Figure 3.**
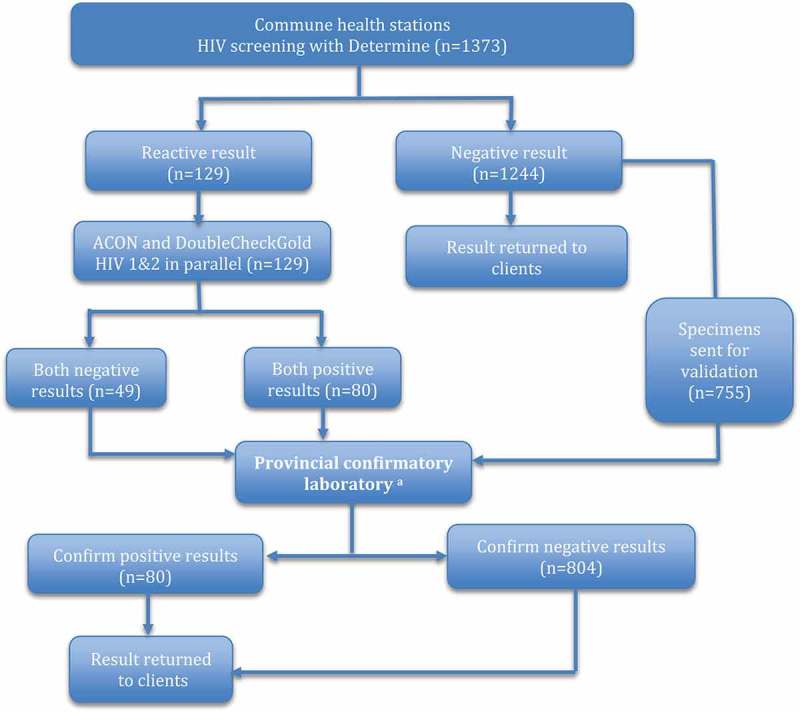
HIV testing validation procedure. ^a^ algorithm used at confirmatory testing:Murex HIV Ab/Ag Combination or Genscreen Ultra HIV Ag-Ab, Serodia HIV1/2 and SD Bioline HIV-1/2 3.0 or Determine HIV-1/2

Of the 1,244 specimens that gave a negative screening test result at the CHSs, 755 were sent to the PCL for validation testing along with all 129 specimens that were reactive with the screening test using Determine (Figure 3). At the PCLs, all negative specimens sent from CHS were confirmed as negative by Genscreen or Murex EIA. All 80 specimens recorded as positive at CHS using the three rapid test algorithm were confirmed as positive by the algorithm in use at the supervising PCL (Table 3). Forty-nine specimens that were reactive with Determine but negative with ACON and DoubleCheckGold at the CHSs were confirmed negative by the PCL (Table 4). Based on the results in Table 3, sensitivity and specificity of this rapid test algorithm was calculated as below:

**Table 3.** Comparison of HIV test results using the rapid HIV testing algorithm at CHSs with test results using an ELISA-based testing algorithm at the provincial confirmatory laboratories ^a^

	Results at provincial confirmatory laboratory (considered to be the gold standard)		
Testing results at CHSs using three rapid test algorithm	Positive	Negative	Total
HIV positive (reactive with all three rapid tests)	80	0	80
HIV negative (negative with Determine)	0	755	755
**Total**	**80**	**804**	**884**

^a^ This table excluded 49 specimens which had indeterminate results (reactive with Determine but negative with ACON and DoubCheckGold)

**Table 4.** Rate of falsely reactive Determine test results by risk group

Testing populations	False reactive	%
Pregnant women	31	63.3
PWID	4	8.2
Partners of PLHIV or PWID	10	20.4
Other	4	8.2
Total	49	100.0

Sensitivity = 80 /(80 + 0)*100 = 100%; Specificity = 755 /(0 + 755)*100 = 100%

### Performance of pilot sites in external quality assessment schemes

All 21 CHSs participated in EQA provided by NIHE. In 2013, 21 CHSs received two rounds of EQA panels. In the first round, 20/21 CHS submitted accurate results whereas in the second round 20/20 provided accurate results. (One CHS did not submit their results to NIHE for round 2).

## Discussion

At the time of this pilot study, HIV confirmatory testing in Vietnam was still centralized at provincial health facilities and HIV screening was only available at the district facilities. The national HIV testing policy at that time required that the testing algorithm to confirm a specimen as HIV positive included an EIA. As a result, only provincial laboratories, where the equipment and expertise to perform an EIA were available, provided confirmatory HIV testing services. Limited availability of screening and confirmatory HIV testing services and the need for an EIA for confirmatory testing contributed to low HTC uptake among key populations, long turnaround time for test results and loss of clients to follow-up after the HIV testing. For instance, in mountainous remote settings, less frequent specimen transportation from the district to the PCL contributed to increased turnaround times. A study in one mountainous province found that it took 37 days on average (ranged from 6–131 days) from the time HIV was screened at HIV screening sites until the sites received confirmatory results from PCL [8]. Similarly, in PCLs with low throughput, a requirement to test the specimens in batches using EIAs to maximize efficiency caused unacceptable delays in turnaround time.

This pilot study demonstrated a feasible and sustainable model for decentralization of HIV confirmatory testing from the provincial level down to the commune level. In the current health system in Vietnam, the CHS is the grass root level of healthcare. Prior to the pilot study, commune health staff provided primary healthcare for people living in their commune. However, HIV services were not provided at the commune level. Thus, in geographically difficult settings such as Dien Bien province, access to HIV services including HTC was very challenging for many people. In this pilot study, integration of HIV testing into the primary healthcare system brought HTC services close to peoples’ homes, which facilitated better access to HTC. The Treatment 2.0 evaluation report strongly suggested high acceptability and appreciation of PLHIV for being able to access HTC service at CHS due to its convenience and time-saving (unpublished data). In addition, using the existing healthcare system to provide HIV testing services is critical for Vietnam to sustain its HIV programme in context with the decline in funding from external sources. Following this pilot, Vietnam has been piloting “test for triage” recommended by WHO 2015 consolidated guidelines on HIV testing services [9] to further decentralize HTC services at the community level. In this pilot, village health workers and peer educators in selected communes were trained to provide HIV screening tests and linkage to care. The results will be published in a separate paper. In addition to improving access to HIV testing for key populations, access to HIV testing among pregnant women was also increased since both HTC and ANC services were offered at CHS. Vietnam is aiming to eliminate mother to child transmission of HIV by 2020 [10] and this target can only be achieved if at least 90% of pregnant women know their HIV status. Although it will need investment from the government, it has been shown to be cost-effective even in low prevalence setting [11].

The results from this pilot study also suggested that an appropriately validated rapid testing algorithm, the provision of training and the access to quality assurance programmes can be used at primary healthcare settings allowing confirmation of HIV positive results without compromising the accuracy of HIV testing. The data from this pilot study has shown consistent results between the rapid test algorithm performed by commune health staff and the more sophisticated algorithms used in provincial laboratories. This suggests that it is possible to decentralize HIV confirmatory testing to commune health facilities in Vietnam to facilitate early access to diagnosis and treatment for PLHIV, their partners, key populations and pregnant women. The evaluation of the Treatment 2.0 pilot data, revealed that people diagnosed with HIV at communes in Dien Bien started ART at a higher CD4 count (median 294 cells/mm^3^) than those who were diagnosed at district facilities (median 88 cells/mm^3^) [12]. Experiences in other countries also indicate that using a rapid testing algorithm could optimize HTC service delivery and improve linkage to care [13,14].

This pilot also mobilized and trained village heath workers and peer educators to reach out to key populations and facilitate linkage between diagnosis, care and treatment. Although more than 80% of people diagnosed with HIV were successful linked to OPC for care and treatment, five people died during this pilot suggesting late diagnosis and treatment need to be addressed by making testing more accessible and strengthening linkage between diagnosis and treatment and care to ensure people diagnosed with HIV received ART in timely manner. Additionally, a mechanism for tracking clients’ needs to be developed to follow-up with clients who do not return for their results.

The study findings suggested the rapid HIV testing algorithm could be applicable to larger programmes with consideration to establishing a functioning system to ensure a high quality of testing and minimize misdiagnosis. The fact that no discordant results were identified in the pilot study is most likely the result of well trained staff, availability of regular technical support and supervision of testers, participation in an EQA scheme and use of a well validated testing algorithm. It was also noted that one of 21 piloting CHS reported one or more aberrant results in one EQA round and another CHS did not report results for one EQA round. This suggested incorrect result could happen at commune facilities and thus regular technical assistance and monitoring is required to ensure quality assurance measures are effective. In this pilot, with strong supervision systems in place, the CHS that reported an aberrant result in EQA was provided with assistance to rectify the problem. In addition, verification of HIV status before ART initiation should be considered by policy makers especially in the context of expansion of decentralization of HIV confirmatory testing to primary healthcare facilities to prevent misdiagnosis and mistreatment as recommended by WHO 2016 [15]. The testing procedure implemented in this pilot was slightly different from the WHO recommended strategy for HIV diagnosis. In this pilot, assay 2 and 3 were performed in parallel if assay 1 was reactive. The reason for this is that this is the first time ever that HIV testing had been introduced at the commune health stations, who had no previous testing experience. Thus, we try to simplify the testing procedure by performing the second (A2) and third (A3) assays in parallel. Commune health staff were trained to record a positive result only if all three assays were reactive. Any discordant results between the three assays were recorded as indeterminate and required confirmation by the PCL.

In a larger Nigerian study, where a two rapid test algorithm was used, 6% of HIV test results were reported falsely positive. The authors suggested potential risks for errors including lack of a quality management system in these laboratories, inconsistent or incorrect use of rapid test algorithms; incorrect interpretation of the weakly-positive test lines and non-usage of a third test [16]. At a WHO meeting held in Geneva in March 2016 discussing the social, public health, human rights, ethical and legal implications of misdiagnosis of HIV status, WHO and the US Centers for Disease Control reported a range of 0.7–10.5% misdiagnosis of HIV-positive status from programme settings and external quality assessment schemes [17]. Common issues accounting for misdiagnosis raised in this meeting included difficulty in reading weakly reactive lines, not using a validated national testing algorithm or a WHO recommended testing strategy, poor training, support and supervision of testers, specimen mix-up and not following standard operating procedures [18–20]. Therefore, to expand community-based HIV testing in Vietnam using a rapid testing algorithm, a quality management system needs to be well established within the programme including a standardized training curriculum, standard operating procedures, technical assistance and supervision and an EQA scheme. Furthermore, the national testing algorithm needs to be implemented at all testing sites.

This pilot study has several limitations. First, this pilot was designed as a demonstration pilot to assess feasibility including quality of HIV POC diagnosis at commune health facility and not designed for validation of the testing algorithm, thus sample size may not be large enough to conclude sensitivity and specificity of the testing algorithm. The pilot was implemented in only two provinces, one mountainous province in the North West and one city in Mekong River Delta. Thus, in other provinces with relatively different culture, geographical characteristics and level of stigma and discrimination, willingness to access HTC at commune health stations among key populations may not be the same and interventions need to be tailored to meet the needs of these key populations to enhance the efficiency of HIV POC diagnosis.

## Conclusions

Decentralization of HIV confirmatory testing to CHS is feasible in Vietnam. The results obtained using the rapid testing algorithm provided strong evidence on the feasibility of HIV testing at primary healthcare settings. Quality assurance measures including training, competency assessment, regular monitoring and supervision and an EQA scheme are essential to ensure accurate test results. This pilot made an important contribution by providing data that convinced the Ministry of Health to amend its policy to decentralize HIV testing services and to ensure high-quality HIV testing is available at primary healthcare facilities.
